# OneG: A Computational Tool for Predicting Cryptic Intermediates in the Unfolding Kinetics of Proteins under Native Conditions

**DOI:** 10.1371/journal.pone.0032465

**Published:** 2012-03-07

**Authors:** Tambi Richa, Thirunavukkarasu Sivaraman

**Affiliations:** Department of Bioinformatics, School of Chemical and Biotechnology, SASTRA University, Thanjavur, Tamil Nadu, India; University of South Florida College of Medicine, United States of America

## Abstract

Understanding the relationships between conformations of proteins and their stabilities is one key to address the protein folding paradigm. The free energy change (ΔG) of unfolding reactions of proteins is measured by traditional denaturation methods and native hydrogen-deuterium (H/D) exchange methods. However, the free energy of unfolding (ΔG_U_) and the free energy of exchange (ΔG_HX_) of proteins are not in good agreement, though the experimental conditions of both methods are well matching to each other. The anomaly is due to any one or combinations of the following reasons: (i) effects of *cis-trans* proline isomerisation under equilibrium unfolding reactions of proteins (ii) inappropriateness in accounting the baselines of melting curves (iii) presence of cryptic intermediates, which may elude the melting curve analysis and (iv) existence of higher energy metastable states in the H/D exchange reactions of proteins. Herein, we have developed a novel computational tool, OneG, which accounts the discrepancy between ΔG_U_ and ΔG_HX_ of proteins by systematically accounting all the four factors mentioned above. The program is fully automated and requires four inputs: three-dimensional structures of proteins, ΔG_U_, ΔG_U_^*^ and residue-specific ΔG_HX_ determined under EX2-exchange conditions in the absence of denaturants. The robustness of the program has been validated using experimental data available for proteins such as cytochrome c and apocytochrome b_562_ and the data analyses revealed that cryptic intermediates of the proteins detected by the experimental methods and the cryptic intermediates predicted by the OneG for those proteins were in good agreement. Furthermore, using OneG, we have shown possible existence of cryptic intermediates and metastable states in the unfolding pathways of cardiotoxin III and cobrotoxin, respectively, which are homologous proteins. The unique application of the program to map the unfolding pathways of proteins under native conditions have been brought into fore and the program is publicly available at http://sblab.sastra.edu/oneg.html

## Introduction

Each protein adopts a specific well-defined three-dimensional (3D) structure, which is important for its biological activities. The relationships between the conformations of such proteins and their stabilities have intrigued researchers for many decades [1]. The conformational stabilities indicate the free energy differences between the folded (N) and the unfolded conformations (U) of proteins. In general, the stabilization free energies of protein molecules have been determined from the studies of protein unfolding caused by denaturants and temperature [2]. Under a reversible and two-state unfolding process, the population of ‘N’ and ‘U’ of a protein could be precisely estimated by using optical techniques such as fluorescence spectrometry and circular dichroism. The free energy of unfolding (ΔG_U_) of proteins is calculated by fitting their unfolded population (U) plotted with respect to denaturant concentration or temperature, to an appropriate two-state model equation [3]. The classical melting analyses provide clues on understanding the mechanism of unfolding (two-state/multi-state processes) and the 3D structural architectures (domains organization) of proteins [4]. Hence, estimation of an accurate ΔG_U_ (free energy of unfolding) for proteins at ambient conditions is indispensable to unambiguously address the thermodynamic and kinetic events of the proteins.

Residue-specific free energy changes of proteins have been determined under native conditions by using hydrogen-deuterium (H/D) exchange method in conjunction with NMR technique [5]. In a typical H/D exchange experiment, when a protein is dissolved in deuterium oxide (D_2_O), backbone amide protons (NHs) of the protein begin to exchange with deuterium. The H/D exchanges of the NHs can be interpreted with the two-state model proposed by Hvidt [6]. In the model, Closed (NH) and Open (NH) represent folded and unfolded conformations of proteins, respectively.
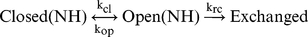
(1)The rate constants k_op_ and k_cl_ are for the unfolding and the refolding reactions, respectively. Exchange takes place only from the unfolded state with the rate constant of k_rc_, which can be predicted using the method reported by Bai *et al.* [7]. Under EX2 exchange conditions, where k_cl_ is many-folds greater than k_rc_, k_ex_ (exchange rate constants of amide protons) is defined as:
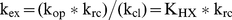
(2)where K_HX_ is the residue-specific equilibrium constant for NHs in proteins. The residue-specific free energy is then determined using the following relationship.
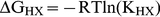
(3)where R is the gas constant and T is the absolute temperature. The free energy of exchange of protein is averaged out to four largest residue-specific ΔG_HX_ of the protein [8], [9].

When the ΔG_HX_ (estimated from H/D exchange method) and the ΔG_U_ (determined from melting curves) are true representations of global unfolding events of the proteins, both the parameters of the proteins must be in good agreement under similar experimental conditions. Contrary to the expectation, proteins show strong discrepancies between their ΔG_HX_ and ΔG_U_ values. If the discrepancy is smaller, it can be well accounted to the effect of *cis-trans* proline isomerisation and/or to the effect of baselines of melting curves [8], [9], [10], whereas larger differences between ΔG_HX_ and ΔG_U_ are attributed to the possible existence of cryptic intermediates accumulating in the equilibrium unfolding reactions of proteins under native conditions [11], [12]. The cryptic intermediates elude the melting curve analyses of proteins as they are short-lived and unstable and hence, the intermediates cause underestimation of the ΔG_U_. However, the cryptic intermediates can be qualitatively detected by denaturant-dependent H/D exchange method in conjunction with NMR technique [11], [12]. In this method, exchange rates (k_ex_) of NHs of proteins are measured at low concentrations of denaturant, which only affect the equilibrium between folded and unfolded conformations of proteins. Notwithstanding the advantages of detecting cryptic intermediates and residues constituting each intermediate from such studies, the methods are laborious, expensive, time consuming and also requires sound experimental knowledge. Proteins that are not accumulating cryptic intermediates in their unfolding pathways may also depict discrepancies between the ΔG_HX_ and the ΔG_U_ and the discrepancy may probably due to the existence of metastable states causing overestimation of the ΔG_HX_. Detecting exact metastable states of proteins is a challenging experimental task of protein folding [13]. In this context, computational methods will be an excellent alternative to address the possible existence of cryptic intermediates/metastable states in the unfolding events of proteins under native conditions. To date, there were no unique programs to address the above mentioned discrepancies in the stabilities of proteins, to our best knowledge. However, it should be mentioned that there are several programs to predict exchange rates of amide protons of proteins and also to predict the folding/unfolding rates of proteins from their amino acid sequences. For instance, programs such as SPHERE [14] and CamP [15] are predicting the k_rc_ and the protection factors of NHs in proteins from their amino acid sequences, respectively. Dovidchenko *et al.* [16] have recently described a method on prediction of amino acid residues protected from H/D exchange in a protein chain. Wolynes *et al.* used a statistical approach to figure-out the energetic of protein conformations and relative foldability for contagious segments present in proteins [17], [18]. RAFT (rapid autonomous fragment test) program predicts autonomous folding unit based on the analysis of inter-residue contacts of structural segments present in the native structure of proteins [19]. COREX/BEST, which is an interesting program developed by VJ Hilser [20], defines native state ensembles and also maps rigidities and flexibilities of various regions of proteins. In the present study, we have herein developed a computational program, OneG, which predicts possible existence of cryptic intermediates/metastable states of proteins from their 3D structures, ΔG_U_, ΔG_U_^*^ and residue-specific ΔG_HX_ determined under native conditions. The OneG employs ‘contact order matrix’ strategies for all amide protons (NHs) that are hydrogen bonded in regular secondary structural elements of proteins, to achieve the task. The robustness of the program has been validated by predicting cryptic intermediates of proteins such as cytochrome c and apocytochrome b_562_ for which experimentally characterized cryptic intermediates have been well documented in the literature. It is important to point-out that the program does not imply/support for the absence of cryptic intermediates/metastable states in the native unfolding of proteins for which the ΔG_U_ and ΔG_HX_ are in good agreement (this aspect is beyond the scope of the article). Similarly, there is no straightforward correlation between the accumulation of cryptic intermediates of proteins under native conditions and the free energy discrepancies (ΔG_U_ vs. ΔG_HX_) of the proteins. In this background, the applications of the program have also been dealt in detail on understanding the unfolding events of two structurally similar proteins (cardiotoxin III (2CRT) and cobrotoxin (1COD)), under native equilibrium conditions.

## Results and Discussion

### Estimations of k_rc_ and ΔG_HX_ for amide protons (NHs) in proteins

The intrinsic exchange rate constants, k_rc_, for NHs of a protein can be estimated under defined experimental conditions (pH, temperature and ionic strength) on the basis of model compound studies [7], [21] and using the equation-4:
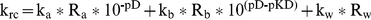
(4)where k_a_, k_b_ and k_w_ are rate constants of acid, base and water catalyzed exchange reactions, respectively; R_a_, R_b_ and R_w_ are the effect of residues that are on the left and the right sides of amide protons under considerations at acidic, basic and neutral conditions, respectively; pK_D_ is the molar ionization constant of D_2_O; pD is the pH-meter reading corrected to deuterium effect. The effect of temperature on the intrinsic exchange rates of the NHs is calculated using the following equation.
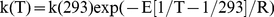
(5)where k(T) and k(293) are rate constants at desired temperature and 293 K, respectively; E is the activation energy and its value for acidic, basic and neutral exchange reactions are 14 kcal/mol/K, 17 kcal/mol/K and 19 kcal/mol/K, respectively; T is the absolute temperature in Kelvin and R is the gas constant. Solving the equations-4 & -5 yields the equation-6 (mathematical derivations not shown).
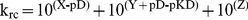
(6)where,
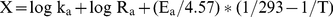
(7)

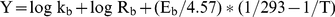
(8)

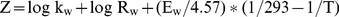
(9)


**Figure 1:**
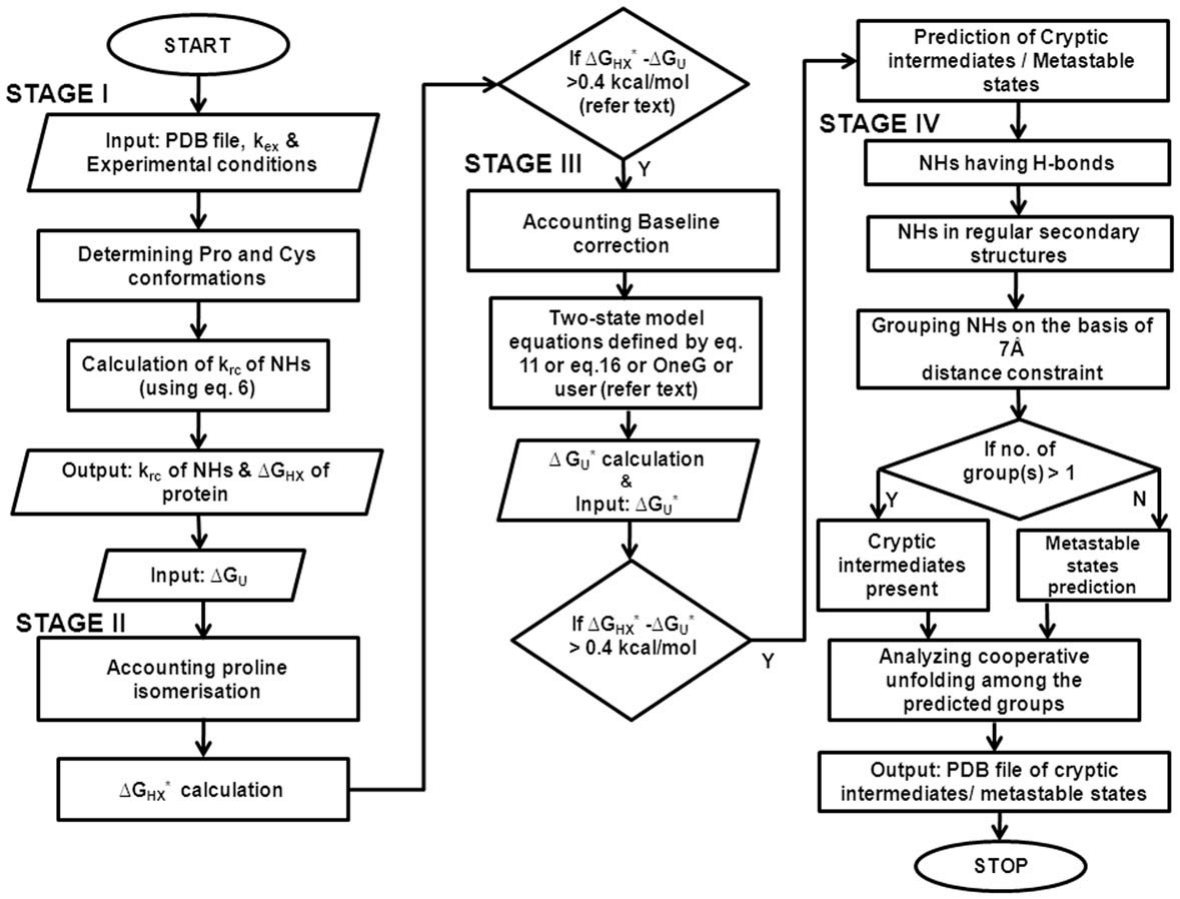
Flowchart of OneG used to predict cryptic intermediates of proteins. Flowchart outlines key-steps used to account the discrepancy between the ΔG_U_ and the ΔG_HX_ and to predict the possible existence of cryptic intermediates/metastable states of proteins.

**Figure 2:**
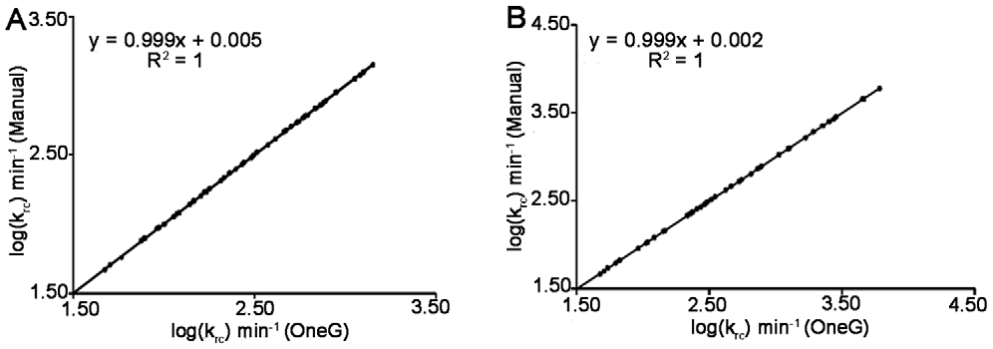
Calculation of k_rc_ of NHs in proteins from their 3D structures. Correlation between k_rc_ values estimated by manual calculation and the OneG program for NHs in proteins (A) Ubiquitin (1UBQ) and (B) Cardiotoxin III (2CRT) at pH 7.0, 298 K.

The OneG calculates the values of k_rc_ for NHs in protein using its PDB file and the equation-6, at defined pH, temperature and ionic strength. Figure 1 outlines the four stages for successfully completing a test run of OneG and the essential steps of stage-I for calculating k_rc_ of NHs are depicted in the Figure S1. Using atomic coordinates of proteins, the program determines disulfide bridges, cysteine residues and geometrical confirmations of Xaa-Pro peptide bonds in proteins and accounts them on calculating the k_rc_ for NHs in the proteins. Any two cysteins in a protein are considered as cystine, when the distance between the two sulphur atoms of the cysteine residues is within 2.3 Å [22] and this particular function of the program has been validated by predicting the cysteine and cystine residues in cardiotoxin III (PDB ID: 2CRT) and cytochrome C (PDB ID: 1HRC, Table 1). The reliability of the program on predicting *cis-trans* proline conformations in proteins is discussed in the next heading. The k_rc_ values of NHs in ubiquitin (PDB ID: 1UBQ) and cardiotoxin III have been calculated using the OneG and the data have been compared with the k_rc_ values of NHs in the proteins as determined by manual calculation (Figure 2). The data in Figure 2A and 2B were fitted to a simple linear equation and the fitted parameters such as slope and positive correlation coefficient were found to be 0.999 and 1.0, respectively, for both proteins. These observations unambiguously demonstrate the reliability of the OneG on calculating the k_rc_ of NHs in proteins. The program, then, calculates residue-specific free energies, protection factors and ΔG_HX_ of proteins with the only requirement of having NMR-derived k_ex_ of NHs in the proteins (estimated under EX2 conditions in the absence of denaturants). Instead of providing residue-specific k_ex_ of NHs, residue-specific ΔG_HX_ (second input) can also be directly given to the program. Upon given the third input of ΔG_U_ (determined from the melting curve analyses of proteins), the program carries forward all the parameters derived in this stage to its second stage for accounting the consistency between the ΔG_HX_, exchange free energy of the proteins and ΔG_U_, unfolding free energy of the proteins.

**Table 1:** Comparison of the actual and the predicted (by OneG program) cysteine and cystine residues in Cardiotoxin III and Cytochrome C.

Sl. No	Protein (PDB ID)	No. of Cys#	Position of Cys	Distance measured by manual method*		Actual Conformation of Cys residue	Distance calculated by OneG program		Predicted conformation of Cys residue
				S-S Pair	Distance		S-S Pair	Distance	
1.	Cardiotoxin III (2CRT)	8	3	C3–C21	2.02	Cystine	C3–C21	2.018	Cystine
			21						
			14	C14–C38	2.01	Cystine	C14–C38	2.013	Cystine
			38						
			42	C42–C53	2.02	Cystine	C42–C53	2.023	Cystine
			53						
			54	C54–C59	2.02	Cystine	C54–C59	2.022	Cystine
			59						
2.	Cytochrome C (1HRC)	2	14	none	8.71	Cysteine	none	8.712	Cysteine
			17						

# Cys denotes Cysteine residue.

*The manual distance measurements for determining the cysteine and cystine residues in proteins were carried-out using PyMol molecular visualization tool [23].

### *cis-trans* isomerisation effect of proline residues on the ΔG_HX_ of proteins

All standard amino acids, except proline, are connected to one another through amide linkages in proteins, whereas proline is linked to the preceding amino acid through an imide bond. The amide bonds are exclusively in *trans* conformations in folded proteins [24]. Contrary to this, the imide bond favours *cis* or *trans* conformations much more equally as the free energy differences between these two conformers are insignificant in proteins [25]. Similarly, amide bonds prefer negligible percentage of (about 0.03%) *cis*-conformations in the unfolded states, whereas imide bond (Xaa-Pro) prefers remarkable percentage of *cis*-conformations in the unfolded states and the percentage varies (6–38%) depending on the chemical properties of the residue (Xaa) preceding proline [24]. When a protein having *cis*-prolines is unfolded by denaturants, the unfolding process of the protein is probably a three-state reaction under equilibrium conditions as shown below:
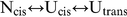
(10)where N_cis_ is the folded protein with proline residues in *cis*-conformation; U_cis_ and U_trans_ are the unfolded protein with proline residues in *cis* and *trans* conformations, respectively. The free energy change (ΔG_U_) of the protein estimated by denaturation method accounts the equilibrium constants of both steps in the reaction. But, H/D exchange method determines equilibrium constant for the first step only, since *cis-trans* proline isomerisation is a slow process under native conditions. Due to which, the ΔG_U_ determined by optical methods is usually less than the ΔG_HX_ determined by H/D exchange method. The ΔG_HX_ corrected to the effect of the *cis-trans* proline isomerisation is denoted as ΔG_HX_^*^, which can be readily calculated using methods reported by Huyghues-Despointes *et al*. [9]. The ΔG_U_ and the ΔG_HX_^*^ of a protein will be in good agreement when the discrepancy between the ΔG_U_ and ΔG_HX_ is merely due to *cis-trans* isomerisation of proline residues present in the protein.

In order to estimate the effect of *cis-trans* isomerisation of proline residues in proteins, the OneG program uses a bee-line for the calculations as shown in the Figure 1 and detailed steps of the stage-II of the program are shown in the Figure S2. The program first determines number of proline residues and their conformations in a protein using PDB file of the protein itself. The reliability of the program on predicting *cis-trans* proline conformations in proteins such as ubiquitin, RNase A (PDB ID: 5RSA) and cardiotoxin III is depicted in Table 2. A quick inspection to the table suggests that the OneG program predicts the exact conformations of proline residues in the proteins. The OneG calculates ΔG_HX_^*^ of proteins based on the K_Pro_ values derived from the model compound studies to all twenty types of Xaa-Pro and the values are stored as default parameters in the program. Table 3 lists ΔG_HX_ and ΔG_U_ reported in the literature for 16 different proteins along with their ΔG_HX_^*^ calculated by the OneG program. The discrepancies between the ΔG_HX_ and ΔG_U_ of the proteins that are listed under ‘Group I’ could be well accounted by proline isomerisation effect alone, since the ΔG_HX_^*^ and ΔG_U_ of those proteins are in good agreement within the tolerance level of 0.4 kcal/mol [8], [9]. Contrary to these observations, the discrepancies between ΔG_HX_ and ΔG_U_ of the proteins listed under ‘Group II’ could not be addressed by proline isomerisation effect alone. This finding implies that the discrepancies may probably stem from different origins that need to be identified and accounted not only for determining the exact ΔG (change in free energy) of the proteins and also to understand the correlations between the kinetic and thermodynamic unfolding events of the proteins. The discrepancy may also be originated due to the default consideration of K_pro_ values from the model compounds in the program. It is possible that the K_Pro_ estimated based on the model compounds in a set of particular experimental conditions may not be a true representation to Xaa-Pro of a protein in a totally different solution conditions. For instance, Tyr(92)-Pro(93) of RNase A was found to have 33% *cis* in heat unfolded states of the protein [37], which largely differs from predicted percentage (24%) of *cis* conformations for the imide bond. However, values of K_pro_ estimated from the model compounds account reasonably the *cis-trans* isomerisation of Xaa-Pro peptide bonds in the unfolded states of most proteins [9], [24], [25]. Thus, the OneG program calculates ΔG_HX_^*^ of proteins using the K_Pro_ obtained from the model compounds, by default. However, the program provides an option to use K_Pro_ determined from the studies on proteins for calculating the ΔG_HX_^*^. The program then compares the ΔG_HX_^*^ of proteins with their ΔG_U_ and carries forward the values of ΔG_HX_^*^, ΔG_HX_ and ΔG_U_ to the next stage of the program for further calculations.

**Table 2:** Comparison of the actual and the predicted conformations (by OneG program) of Xaa-Pro peptide bonds in Ubiquitin, Rnase A and Cardiotoxin III.

Sl. No	Protein (PDB ID)	No. of Prolines	Position of Prolines	Distance measured by manual method*		Actual Conformation of Xaa-Pro peptide bond	Distance calculated by OneG program		Predicted conformation of Xaa-Pro peptide bond
				C_α_-C_α_	C_α_-C_δ_		C_α_-C_α_	C_α_-C_δ_	
1.	Ubiquitin (1UBQ)	3	19	3.81	2.88	***Trans***	3.811	2.878	***Trans***
			37	3.83	2.85	***Trans***	3.829	2.852	***Trans***
			38	3.81	2.96	***Trans***	3.812	2.961	***Trans***
2.	RNase A (5RSA)	4	42	3.86	2.90	***Trans***	3.858	2.898	***Trans***
			93	3.04	3.88	***Cis***	3.039	3.883	***Cis***
			114	2.91	3.82	***Cis***	2.912	3.817	***Cis***
			117	3.83	2.88	***Trans***	3.828	2.878	***Trans***
3.	Cardiotoxin III (2CRT)	5	8	3.85	2.80	***Trans***	3.847	2.803	***Trans***
			15	3.79	2.76	***Trans***	3.792	2.763	***Trans***
			30	3.82	2.79	***Trans***	3.816	2.787	***Trans***
			33	3.80	2.76	***Trans***	3.802	2.759	***Trans***
			43	3.81	2.82	***Trans***	3.811	2.818	***Trans***

*The manual distance measurements for determining the conformations of the Xaa-pro peptide bonds in proteins were carried-out using PyMol molecular visualization tool [23].

**Table 3:** The values of ΔG_U_, ΔG_HX_ and ΔG_HX_* (free energy of exchange corrected to effect of *cis-trans* proline isomerisation) of sixteen different proteins are herein listed.

Sl. No.	Proteins@	ΔG_HX_	ΔG_HX_*	ΔG_U_	(ΔG_HX_* - ΔG_U_)
**GROUP I**					
1	OMTKY3 [26]	8.2	7.2	7.2	0
2	Barnase [27]	10.1	9.9	9.8	0.1
3	CI2 [28]	7.6	7.1	7.0	0.1
4	434cro [29]	4.0	3.9	3.7	0.2
5	RNase T1 [9]	10.7	8.2	7.9	0.3
**GROUP II**					
6	HEWL [30]	12.4	12.2	11.7	0.5
7	RNase H (E. coli) [9]	10.9	9.3	9.9	0.6
8	Barstar [31]	6.2	6.0	5.0	1.0
9	HPr (E. coli) [9]	5.8	5.7	4.7	1.0
10	Src SH3 domain [32]	6.2	6.1	4.7	1.4
11	CBTX [33]	3.9	3.8	2.3	1.5
12	T4 Lysozyme [34]	17.7	17.5	16.0	1.5
13	Apocytochrome b562 [35]	5.5	5.3	3.3	2.0
14	CTX III [33]	6.6	6.3	4.2	2.1
15	PPL [36]	7.0	7.0	4.9	2.1
16	Cytochrome c [11]	13.0	12.7	10.0	2.7

@ Parentheses contain references from which the values of the free energies of the proteins have been referred.

The values of ΔG_HX_* of the proteins have been calculated using the OneG program. Free energy values of the proteins were represented in kcal/mol.

### Baselines effect of melting curves on the estimation of the ΔG_U_

Denaturant-induced unfolding of proteins under equilibrium conditions is generally monitored by optical probes for estimating the ΔG_U_ of the proteins [38], [39]. In a typical all-or-none unfolding experiment, the observed signals representing the ratio of folded and unfolded states of proteins are plotted with respect to denaturant concentrations. The data are then fitted to the non-linear least squares equation-11 proposed by Santoro and Bolen for estimating the ΔG_U_ of proteins [40].
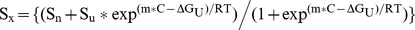
(11)where, S_x_ is the observed signals at various concentrations of denaturant, C is the concentration of denaturant in molarities, ΔG_U_ is the free energy of unfolding in the absence of denaturant, m is slope of a plot depicting ΔG_U_ versus concentrations of denaturant, S_n_ and S_u_ are the signals of the folded and the unfolded states of proteins in the absence of denaturant, respectively. The S_n_ and S_u_ are further defined to be linear with respect to denaturant concentrations as shown in equations-12 and -13, respectively.
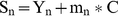
(12)

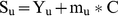
(13)wherein, Y_n_ and Y_u_ are intercepts; m_n_ and m_u_ are slopes; the subscripts ‘n’ and ‘u’ denote the folded and the unfolded conformations, respectively. The equation-11 treats the pre- and post-baselines of the melting curve to be linear with respect to denaturant concentrations. The equation will underestimate or overestimate the ΔG_U_ of proteins, when the baselines of the melting curves of proteins deviate from their linear predictions. It has been shown that when the pre- and post-transitions baselines of heat-induced denaturation of lysozyme were treated by non-linear equations, the change in enthalpy (ΔH) of the protein estimated by optical and calorimetric methods were in good agreement [10].

**Figure 3:**
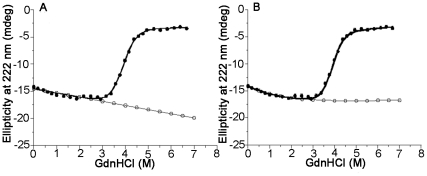
GdnHCl-induced changes in 222 nm ellipticity in ubiquitin in the far-UV region. Solid line through the data in ‘A’ was the fit to the equation-11 and in ‘B’ to the equation-16. Pre-transition baselines were extrapolated using fitted-parameters up to 7 M GdnHCl (refer text).

We emphasize the effect of baselines of melting curves on the estimations of the ΔG_U_ of ubiquitin from its GdnHCl-induced denaturation profile, herein. Figure 3 shows the equilibrium denaturation curve obtained for ubiquitin dissolved in D_2_O at pH 7.0, 288 K, using GdnHCl as the denaturant. The data in Figure 3A have been fitted to the equation-11 using Kaleidagraph software (Synergy Software) and the fitted parameters were as follows: values of ΔG_U_, m and C_m_ were 7.5 kcal/mol, 3.87 kcal/mol/M and 1.94 M, respectively; C_m_ is the concentration of denaturant at which the protein is half-unfolded. Inspection to the Figure 3A indicated that the changes in the ellipticity at 222 nm of the folded protein were in opposite direction to the unfolding transitions, at low concentrations of denaturant (from 0 to 2 M). It suggested that the action of the denaturant on the protein molecules was not in a linear fashion. In these circumstances, fit of the data to the equation-11 would definitely underestimate the ΔG_U_ of the protein molecules. Hence, the pre-transition baseline of the data may presumably be treated by an exponential function shown in the following equation.
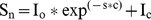
(14)where, ‘s’ is the concentration of denaturant at which changes in optical signals of the folded molecules begin to assume a linear fashion with respect to denaturant concentrations; ‘c’ is the concentration of denaturant; ‘I_c_’ is an asymptote; ‘I_o_’ is amplitude, which is further defined by the following equation:
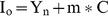
(15)By substituting the equations-14 &-15 in the equation-11, the following equation is derived.
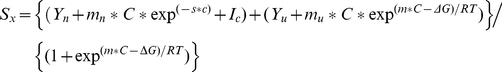
(16)


**Figure 4:**
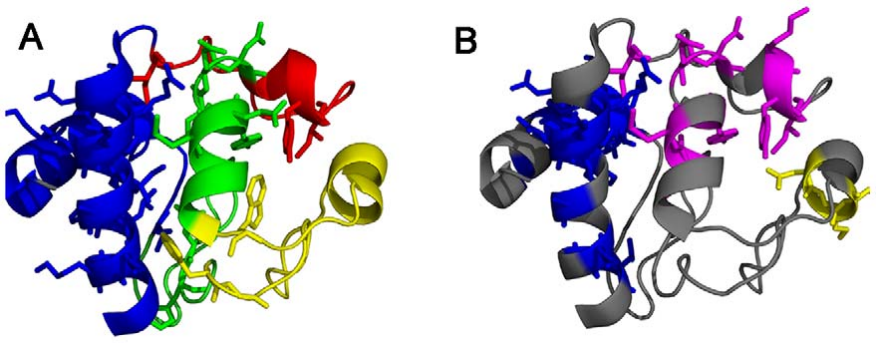
Figurative representation of cryptic intermediates of Cytochrome C. The cryptic intermediates detected by experimental methods and predicted by OneG are shown in Figure 4A and Figure 4B, respectively. The backbone structures of the protein and residues representing each intermediate are shown in ribbon and stick models, respectively. Figure 4A shows cryptic intermediates, proposed on the basis of experimental methods, in blue, green, yellow and red colours. The residues (for which exchange kinetics were observed by experiments) representing each intermediate are shown in sticks. Figure 4B shows residues constituting three distinct intermediates as predicted by OneG program, in blue, magenta and yellow colours.

The fit of the data in the Figure 3B to the equation-16 yielded the following fitted parameters: values of ΔG_U_, m and C_m_ were 8.6 kcal/mol, 3.91 kcal/mol/M and 2.20 M, respectively. The pre-transition baselines of the data in Figure 3A and Figure 3B have been extrapolated up to 7 M of denaturant concentrations using the fitted parameters obtained by treating the data to the equations-11&-16, respectively. When the data were treated by the equation-11, the ellipticity at 222 nm representing folded proteins was linearly changing with respect to denaturant concentrations and consequently the population of unfolded species in the melting region of the curve was overestimated (Figure 3A). In contrary, when the data were treated by the equation-16, the ellipticity representing folded proteins was constant especially in the melting region (Figure 3B) and consequently the equation may estimate exact population of the unfolded species in the region. The difference in the values of ΔG_U_ obtained by fitting the melting curve to the equations-11&-16 was 1.1 kcal/mol. Strikingly, ΔG_U_^*^ (8.6 kcal/mol) of ubiquitin by fitting its melting curve to the equation-16 was in good agreement with the ΔG_HX_^*^ (8.8 kcal/mol) of ubiqutin reported at similar experimental conditions [41]. Thus, it is obvious that pre- and post-transition baselines of melting curves can obscure the accurate determination of the ΔG_U_ of proteins, when they are not treated by suitable equations. OneG program offers three functions (linear, exponential and polynomial) to account the effect of pre- or/and post- transition baselines of melting curves of proteins and constructs non-linear equations as per the options chosen (Figure 1 and Figure S3, which outlines the detailed steps of the stage-III of the program). The equations can be used to fit the melting data for determining the free energy of unfolding of proteins as explained above. The resultant ΔG_U_ of the proteins is denoted as ΔG_U_* in the present study. The OneG, then, compares the ΔG_U_* with the ΔG_HX_* of the proteins. The two values must be in good agreement for proteins for which the equilibrium unfolding pathways are all-or-none (typical two-state) process.

### Predicting cryptic intermediates/metastable states in the unfolding kinetics of proteins under native conditions

If there is a remarkable difference between the ΔG_HX_^*^ and ΔG_U_^*^ of a protein, the origins of the discrepancies may probably stem from many facets. The following factors must be seriously considered to avoid any spurious data from the experiments: (i) experimental conditions such as solvents (H_2_O/D_2_O) and buffer solution must be identical in both melting analysis and H/D exchange methods [9], [38], [39] (ii) the melting curve must be adequately defined with sufficient data points [38], [42] (iii) denaturant concentrations must be accurately determined using refractive index method [39], [43] (iv) H/D exchange method must be performed under pure EX2-exchage mechanism [9]. While the experimental conditions of both optical and H/D exchange methods are matching well to each other, the discrepancies arising between the ΔG_U_ and the ΔG_HX_ of proteins must be either due to accumulation of cryptic intermediates or metastable states in the unfolding kinetics of proteins under native conditions. Since cryptic intermediates are weakly stabilized, they can easily elude from analysis of melting curves of proteins, which in turn causes underestimation of ΔG_U_. On the other hand, ΔG_HX_ would be overestimated, when the H/D exchange reactions of most slowly exchanging NHs are happening through the metastable states of proteins. The metastable states are, in general, heterogeneous denatured-like ensembles of proteins, which are higher in energy than that of denatured states of the proteins. In order to detect cryptic intermediates, denaturant-dependent exchange rates of NHs of proteins need to be estimated under native conditions, using NMR (nuclear magnetic resonance) spectroscopy. Residue-specific folding (k_f_/k_cl_) and unfolding rates (k_u_/k_op_) of NHs of proteins should be determined using H/D exchange methods in conjunction with NMR and mass spectrometry (MS) techniques in order to explore the energetic ensembles of metastable states of proteins. Using the experimental strategies, cryptic intermediates that are accumulating in the unfolding kinetics of proteins such as cytochrome c [44], apocytochrome b_562_ [35], RNase H [45] have been reported in the literature. Similarly, existence of heterogeneous mixture of denatured-like conformations of OMTKY3 has been shown at residue level resolutions from the comprehensive analysis of H/D exchange data of the protein, derived from NMR and MS techniques [46]. Though these experiments can be used to detect and structurally characterize the cryptic intermediates and metastable states of proteins, the methods are expensive, laborious and prerequisite sound knowledge in protein chemistry.

In the fourth stage, the OneG scans proteins to predict either for cryptic intermediates or metastable states that may exist in unfolding kinetics of the proteins (Figure 1 and Figure S4). On the basis of the 3D structures of proteins, ΔG_U_, ΔG_U_* and residue-specific ΔG_HX_, the program executes its predictions using ‘contact order matrix’ strategies, which have been elaborately discussed in ‘Design and implementation’ section. The program accounts the ΔG_HX_ of all NHs that are participating in the regular secondary structural segments of proteins. This is based on the fact that the NHs that are involving in the formation of H-bonds either at surface areas or loop regions of proteins undergo H/D exchanges through local structural fluctuations [45], [47], [48]. In outline, the program divides the NHs into a few numbers of groups based on distance constraints of 7 Å and contact order matrix (see ‘Design and implementation’). Briefly, NH of any residue in a group will be in contact with NH of, at least, any one residue in the same group within 7 Å and residues in a group will be away from any residues of another group, at least with distance of 7 Å. In other words, each group is distinct from other groups in terms of distance constraints and structural contexts. Hence, the program predicts that each distinct group is the representation of possible cryptic intermediate of proteins. Furthermore, the free energy coverage of each and every group is compared with other groups and groups that are having same free energy coverage are denoted as cooperative units. When the program fails to identify distinct cryptic intermediates (or more than one group) on the basis of contact order matrix, the program, by default, begins searching for possible existence of metastable states of proteins, which may lead to the overestimation of ΔG_HX_. The concept of higher energy denatured states has already been introduced in the discussion of slow *cis-trans* proline isomerisation in the denatured states [9], [24], [25]. Extending this argument, any relaxation of the denatured protein that occurs more slowly than refolding should give rise to higher energy metastable denatured state in the exchange experiment. In other words, the lifetime of the denatured state sampled in exchange experiments may be less than the time required for relaxation of denatured protein to its ground state. Perhaps this relaxation process involves diffusion of a relatively compact set of conformers, crossing from the transition state, to the broader distribution of conformers that are characteristic of the denatured ground state. In order to predict the metastable states of proteins, the program cluster all residues for which ΔG_HX_>ΔG_X_ (refer ‘Design and implementation) and the residues may constitute either a continuum or distinct groups of higher energy metastable denatured states of the protein [46], [49]. As discussed above for the cryptic intermediates, the residues constituting metastable states are grouped and cooperative unfolding among the groups are analysed on the basis of ‘contact order matrix’ and ‘free energy coverage’, respectively. The robustness of the program on predicting the cryptic intermediates/metastable states has been validated using proteins such as cytochrome C, apocytochrome b_562_, cardiotoxin III and cobrotoxin. The agreement between the predicted data and the experimental data of the proteins is depicted in Table 4 and the comparative analyses of each protein are discussed below in detail.

**Table 4:** Structural contexts of cryptic intermediates/metastable states characterized to present in the proteins using experimental methods and/or OneG computational tool.

Protein	NHs having H-bonds in regular secondary structures		No. of cryptic intermediates/metastable states		Structural context of cryptic intermediates/metastable states detected by experiments			Structural context of cryptic intermediates/metastable states predicted by OneG		
	Actual	Predicted	Actual	Predicted	No.	Region	Residues	No.	Region	Residues
Cytochrome C	38	38	4 CI*	3 CI	I	N- and C- terminal	K7 K8 F10 V11 Q12 K13 T19 R91 E92 D93 L94 I95 A96 Y97 L98 K99 K100 A101	I	N- and C- terminal	K7 I9 F10 V11 Q12 K13 C14 A15 H18 R91 E92 D93 L94 A96 K99 A101 T102
					II	60's helix	L32 H33 M65 E66 Y67 L68 E69 N70	II	60's helix and 70's loop	L64 M65 Y67 E69 N70 K73 Y74 I75 I85
					III	Region spanning36–61	F36 G37 W59	III	Region spanning 36–61	N52 K53 N54
					IV	70's loop	Y74 I75 I85			
Apocytochrome b_562_	49	49	3 CI	3 CI	I	Helix II and Helix III	K32 M33 R34 A35 A36 A37 G70 Q71 A75 L76 K77	I	Helix II	V26 K27 D28 A29 L30 K32 R34 L38 D39 A40 Q41 K42 A43
					II	Helix IV	A87 Q88 A89 A90 A91	II	Helix III and Helix IV	L67 L68 V69 G70 Q71 I72 A75 L76 A79 N80 E81 V84 A87 Q88 A89 A90 A91 Q93
					III	Helix I	L14 K15 V16 I17	III	Helix I	E8 L10 N11 N13 L14 K15 V16 I17 E18
Cardiotoxin III	20	20	No ED#	2 CI	Not Applicable			I	Triple-stranded domain	C21 K23 M24 F25 M26 V27 V32 V34 K35
								II	Double-stranded domain	C3 K5 K12
Cobrotoxin	14	14	No ED	MS$	Not Applicable			I	Strands III, IV and V	K26 K27 R28 W29 E38 N53 C55

*CI denotes Cryptic intermediates;

# ED denotes Experimental data;

$ MS denotes Metastable states.

### Cytochrome C

**Figure 5:**
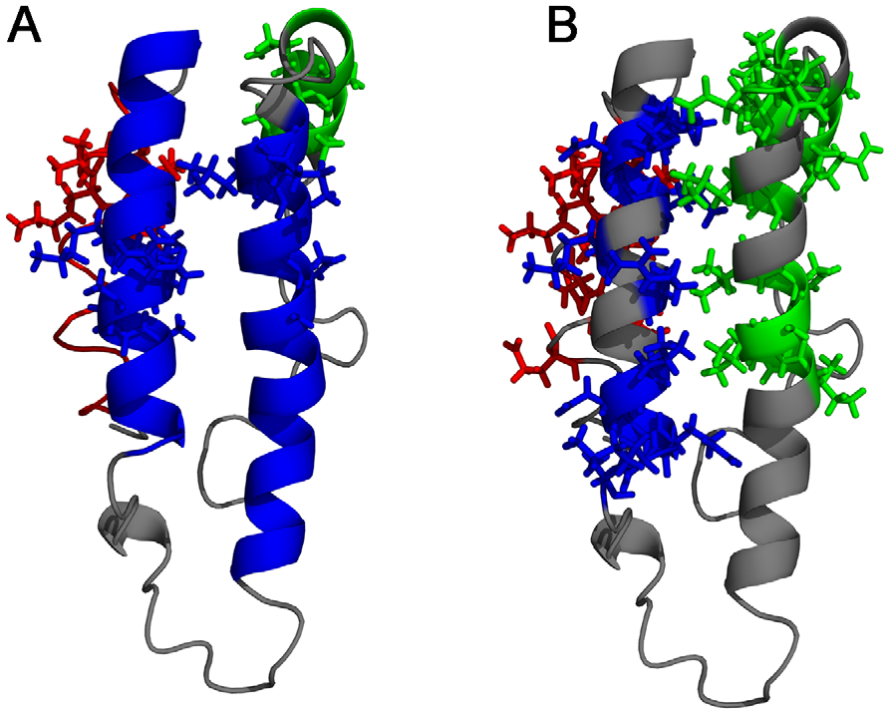
Figurative representation of cryptic intermediates of apocytochrome b_562_. Three cryptic intermediates of the protein detected by experimental methods and predicted by OneG are shown in Figure 5A and Figure 5B, respectively. The intermediates are denoted by blue, green and red colour codes in both cases. The backbone structures of the protein and residues representing each intermediate are shown in ribbon and stick models, respectively. Figure 5A shows residues for which exchange kinetic data were reported in the literature.

Cytochrome c is a simple helical protein consisting of 104 residues. The ΔG_U_ and ΔG_HX_ of the protein were reported as 10.0 kcal/mol and 13.0 kcal/mol, respectively [10]. The discrepancy between the two values was 2.7 kcal/mol, after accounting the effect of *cis-trans* proline isomerisation (Table 3). The discrepancy has been attributed to the existence of four distinct cryptic intermediates that are populating in the unfolding kinetics of the protein as determined by denaturant-dependent H/D exchange in conjunction with NMR techniques [44], [50]. In the original article of the work, the four cryptic intermediates are denoted by four colour codes for sake of clarity: blue consisted of residues from N- and C-termini helices; green consisted of residues from 60's helix and region spanning from 20–35; yellow consisted of residues from the region spanning 36–61; red consisted of residues from the region spanning 70–85 (Table 4). Strikingly, OneG predicted three distinct cryptic intermediates of cytochrome c. First group consisted of residues such as K7, I9, F10, V11, Q12, K13, C14, A15, H18, R91, E92, D93, L94, A96, K99, A101 and T102 from the N- and C- termini helices of the protein and free energy coverage of the group was 4.6–11.2 kcal/mol. This group showed perfect resemblance to the blue cryptic intermediate of the protein detected by the experimental methods. Second group predicted by the OneG consisted of residues (L64, M65, Y67, E69, N70, K73, Y74, I75 and I85) from 60's helix and 70's loop regions of the protein and free energy coverage of the group was 4.5–8.7 kcal/mol. The second group represented the green and red cryptic intermediates together. The program was unable to discriminate the green from red, as the residues from the two regions were within 7 Å, the cut-off distance constraint used in the program. Third group predicted by the program consisted of three residues (N52, K53 and N54), which exactly resembled the yellow cryptic intermediate of the protein. The free energy coverage of the group was 4.5–4.9 kcal/mol. Figure 5 depicts structural contexts of the three cryptic intermediates (Blue, Magenta and Yellow) predicted by the OneG and the four cryptic intermediates (Blue, Green, Red and Yellow) characterized by experimental methods, on the 3D structures of the protein.

### Apocytochrome b_562_

Apocytochrome b_562_ is a monomeric, four helix bundle protein consisting of 106 residues. The ΔG_U_ and the ΔG_HX_ of the protein were reported as 3.3 kcal/mol and 5.5 kcal/mol, respectively [35]. The discrepancy of 2.2 kcal/mol observed between the ΔG_U_ and the ΔG_HX_ of the protein was merely due to the existence of cryptic intermediates of the protein under native conditions [35], [50]. Fuentes and Wand have demonstrated the existence of three distinct cryptic intermediates of the protein and also characterized at structural levels: the first cryptic intermediate consisted of residues from the two central helices of the protein; the second and third cryptic intermediates were comprised of residues from the C-terminal helix and N-terminal helix of the protein, respectively (Table 4). Interestingly, OneG predicts three distinct cryptic intermediates of the protein. The first and third intermediates predicted by the program were composed of residues such as V26, K27, D28, A29, L30, K32, R34, L38, D39, A40, Q41, K42 and A43 from central helices and residues such as E8, L10, N11, N13, L14, K15, V16, I17 and E18 from N-terminal helix, respectively. The predicted first and third intermediates were well resembled with the first and the third intermediates characterized by experimental methods, respectively (Table 4 & Figure 5). However, the second cryptic intermediate predicted by OneG was constituted by residues (I67, L68, V69, G70, Q71, I72, A75, L76, A79, N80, E81, V84) from third helix, which is part of central helical segments and also residues (A87, Q88, A89, A90, A91 and Q93) from C-terminal helix of the protein. This observation is contrary to the second cryptic intermediate characterized by experimental method for the protein, because the intermediate detected by the experiments was constituted by residues from the c-terminal helix only. However, scrutinizing the structural architectures of the protein uncovered that though the third helix is sandwiched by second helix and c-terminal helix, the residues of third helix are much closer to residues from c-terminal helix, vis-à-vis their contacts with residues of second helix. Moreover, the c-terminal helix is a kink-helix as the region connecting the c-terminal helix and third helix of the protein is tightly pulled suggesting the two helices are likely to unfold in a cooperative manner. Since the OneG predicts cryptic intermediates purely on the basis of 3D structures of proteins, the second intermediate of apocytochrome b_562_ predicted by the program is very convincing, though the predicted structures of the intermediate were not exactly same to the experimentally characterized structure of the second intermediate. The free energy coverage of the first, second and third intermediates were 2.83–5.23 kcal/mol, 1.48–4.95 kcal/mol and 2.08–3.42 kcal/mol, respectively.

### Cardiotoxin III (CTXIII)

**Figure 6:**
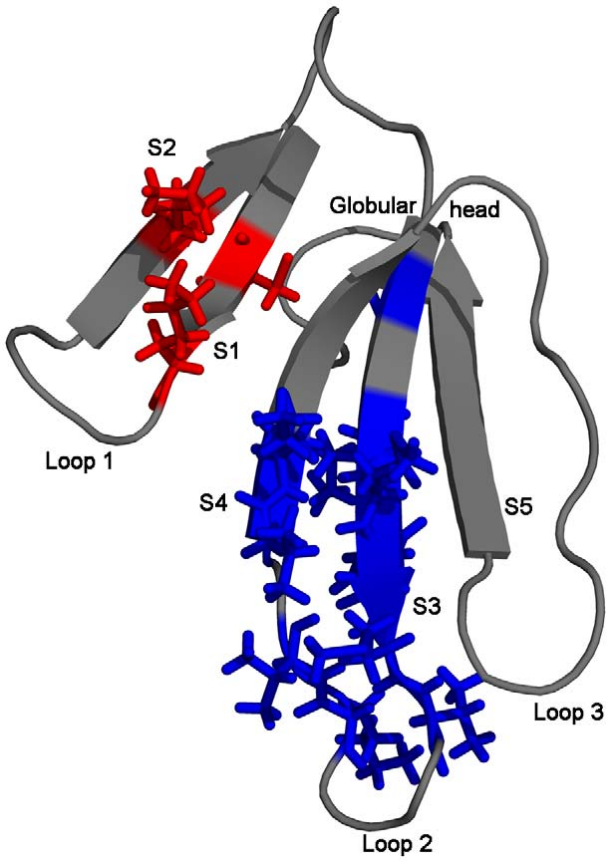
Possible existence of cryptic intermediates of CTX III. The five β-strands (S1–S5), three loops and a globular head in the structure of CTX III (2CRT) are shown by ribbon diagram. The blue and red sticks represent residues in the cryptic intermediates I & II, respectively, as predicted by OneG program.

Cardiotoxin III is monomeric, single polypeptide chain consisting of 60 amino acids and an all β-sheet protein with four disulfide bridges [51]. The ΔG_U_ and ΔG_HX_ of CTX III have been reported to be 4.2 and 6.6 kcal/mol, respectively [33]. After accounting the effects of *cis-trans* proline isomerisation of the protein using the OneG, the recalculated free energy of exchange (ΔG_HX_*) of CTX III was 6.3 kcal/mol (Table 3). The ΔG_U_ and ΔG_U_^*^ of the protein were same, as the chemical denaturation data of CTX III was well fitted to equation-11 [33], [52]. The discrepancy existing between the ΔG_U_ and ΔG_HX_ of the protein has been left unaddressed to date. In order to account the discrepancy, we have herein used the OneG for searching possible existence of any cryptic intermediates of proteins under native conditions, using the 3D structure (2CRT), the ΔG_U_ and the residue-specific ΔG_HX_ of the protein. The program predicts two distinct cryptic intermediates of the protein: the first cryptic intermediate was predicted to be situated in the triple-stranded domain of the protein and was constituted by residues such as C21, K23, M24, F25, M26, V27, V32, V34 and K35; the second cryptic intermediate was composed of three residues (C3,K5 and K12) from the double-stranded domain of the protein (Table 4 and Figure 6). The free energy coverage of the first and second intermediates was 2.43–5.62 kcal/mol and 1.68–4.70 kcal/mol, respectively. Interestingly, the kinetic folding pathways of CTX III have been characterized to proceed through an intermediate accumulating in the burst phase (<5 ms) of the protein [53]. Based on the refolding rate constants of NHs of CTX III obtained from quenched-flow H/D exchange experiments, it has been shown that the triple-stranded β-sheet was formed before the double-stranded β-sheet segment in the refolding kinetics of the protein. Moreover, it has also been demonstrated that the triple-stranded β-sheet segment of the protein was persistently found in the intermediate states identified along the acid-induced and alcohol-induced unfolding pathways of CTX III [54], [55]. To this extent, the predictions of OneG on the possible existence of two cryptic intermediates of CTX III under native conditions are consistent with the data reported from equilibrium and kinetic studies of the protein.

### Cobrotoxin (CBTX)

**Figure 7:**
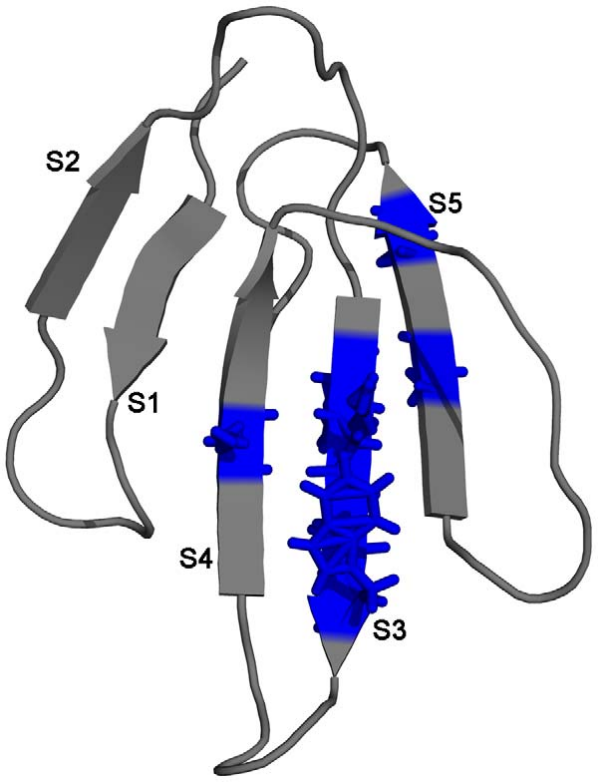
Possible existence of metastable states of CBTX. The overall backbone folding of CBTX (1COD) is shown in ribbon model using PyMol and the five β-strands of the protein are labelled (S1–S5). The residues, predicted by OneG program, constituting the metastable states of CBTX are shown in sticks model.

Cobrotoxin (CBTX) and CTX III are homologous proteins and they are belonging to the three-finger toxin family of *elapidae* snake venoms [56]. The two proteins share high degree of similarities in primary, secondary and tertiary structures to each other [57]. The ΔG_U_ and ΔG_HX_ of CBTX have been reported to be 2.3 and 3.9 kcal/mol, respectively [33], [58]. As the effect of *cis-trans* proline isomersation of the protein accounted only 0.1 kcal/mol and the ΔG_U_^*^of the protein was same as ΔG_U_ [33], the resultant discrepancy of 1.5 kcal/mol was observed between the ΔG_U_ and ΔG_HX_ of the protein and the discrepancy have not yet been addressed, to date. In order to reconcile the discrepancy, OneG was employed as explained in the above sections and the program predicted single cluster consisting of residues from various secondary structural elements of the protein. It implied that there were no possible cryptic intermediates populating in the unfolding kinetics of the protein under native conditions. As the result, the program, by default, attempted to trace for possible existence of metastable states of the protein, with the tolerance limit of 2.4 kcal/mol (ΔG_X_ of CBTX, refer design and implementation). Strikingly, the program predicted a metastable state of the protein, consisting of residues such as K26, K27, W29, R28, E38, N53 and C55, which were dispersed in the strands 3, 4 & 5 of the protein (Table 4 and Figure 7). It has been shown that the chemical unfolding and refolding of the protein proceeded by all-or-none process without the accumulation of intermediates [59]. The kinetic refolding pathways of the protein characterized by chevron plot and using hydrogen-deuterium exchange method in conjunction with multidimensional NMR techniques suggested that a broad continuum of kinetic intermediates, but not distinct intermediates, were populated in the refolding pathways of the protein [59], [60]. To this extent, the OneG prediction for a metastable state of CBTX is in good agreement to the results observed from the equilibrium and the kinetic studies carried-out on the protein. However, it is worthy to mention that the extent of cooperative disruptions of H-bonds in the metastable states of proteins can be unequivocally confirmed by combined NMR and mass spectrometry analyses of H/D exchange of proteins under EX1 conditions [13], [61].

### Concluding remarks

We have herein demonstrated a computational tool, OneG, to address the discrepancy that may arise between the ΔG_U_ and the ΔG_HX_ of proteins, by systematically accounting the following factors: (i) effect for *cis-trans* proline isomerisation (ii) effect of baselines of melting curves on the estimation of ΔG_U_ and (iii) possible existence of cryptic intermediates/higher energy metastable states in the unfolding kinetics of proteins under native conditions. The program prerequisites four inputs, PDB file of proteins, ΔG_U_, residue-specific ΔG_HX_ and ΔG_U_^*^ of the proteins, to successfully complete a test run in a fully automated manner. The robustness of the program has been validated through accounting the discrepancies between ΔG_U_ and the ΔG_HX_ of proteins such as cytochrome c and apocytochrome b_562_ for which experimental rationalizations to reconcile the discrepancies have already been reported in the literature. To our best knowledge, OneG is a unique tool of this kind for systematically analyzing conformational stabilities of proteins. The program is publicly available at http://sblab.sastra.edu/oneg.html. The applications of the OneG program extend beyond rationalizing the conformational stabilities of proteins. The program reveals the degree of cooperative actions among the predicted cryptic intermediates/metastable states. This information may be useful to explore the energy landscapes of the proteins. It is worthy to point-out that several methods have been proposed in the literature to predict rates of folding and rates of unfolding of proteins under defined conditions [62], [63] and consequently, the ΔG_U_ of the proteins can be reasonably calculated. In these connections, developing a tool to predict the residue-specific exchange rate constants at defined conditions (such as pH, temperature, denaturants) on the basis of 3D structures of proteins itself, would also be quite interesting in the near future. The success on the task, in turn, will lead to computationally explore the energetic levels of residues that unfold/refold by various mechanisms (global, sub-global and local structural fluctuations) under native conditions of proteins. Foreseeing the potential applications of the OneG in structural biology, we do anticipate a great scope to improve the software tool at many different aspects.

## Methods

### OneG algorithm

OneG algorithm has been implemented using PERL scripting language [64]. The program accepts both amino acid sequences (represented by single letter codes) and PDB (Protein Data Bank) co-ordinates of proteins for predicting k_rc_ values of NHs. In order to calculate the k_rc_ values, OneG considers temperature in Kelvin, pH in pD (pD = pH+0.4), ionic strength in molarity and activation energies in cal/mol [7], [21]. The values of k_rc_ and ΔG_HX_ for the NHs of protein molecules are expressed in minute^−1^ and kcal/mol, respectively. When a PDB file is the input, the OneG determines the *cis/trans* conformation of Xaa-Pro (Xaa is any one of the twenty standard alpha amino acids and Pro denotes proline) peptide bond in the protein using the following relationship:
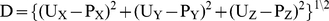
(17)where D is the distance between the C_α_ of Xaa and C_α_ or C_δ_ of proline residue in angstrom; U and P stand for Xaa and Pro residues of Xaa-Pro peptide bond, respectively; X, Y and Z are the atomic co-ordinates of an atom considered. The Xaa-Pro peptide bond is considered as *trans*-conformation, when the distance between C_α_ of Xaa and C_α_ of Pro (C_α_ —C_α_) is greater than the distance between C_α_ of Xaa and C_δ_ of Pro (C_α_—C_δ_). Similarly, when C_α_—C_δ_>C_α_—C_α_, the Xaa-Pro peptide bond is considered as *cis*-conformation [65]. In outline, the program has four stages for each complete cycle. In the first stage, the k_rc_, ΔG_HX_ and protection factors (provided exchange rate constants, k_ex_, are given) of NHs in a protein molecule under consideration are calculated. If the solvent used is other than D_2_O, users need to specify the reference rates the solvent. In the second stage, the ΔG_HX_^*^, the recalculated ΔG_HX_ after accounting for the effect of *cis/trans* proline isomerisation, is compared with ΔG_U_ of the protein molecule. The tolerance level of 0.4 kcal/mol is set as default in the OneG program based on the fact that the more prevalent *trans* form of prolines in proteins contributes about 0.3 kcal/mol to the effect, in general [8], [9]. In the third stage, OneG provides options for fitting the pre- or/and post- transition baselines of melting curves of proteins. The ΔG_U_^*^, the recalculated ΔG_U_ after treating the baselines of melting curve of the protein to an appropriate two-state model equation, is compared with ΔG_HX_^*^ of the protein. In the fourth stage, the program predicts the possible existence of cryptic intermediates/metastable states in the unfolding kinetics of protein on the basis of the 3D structure, ΔG_U_, ΔG_U_^*^ and residue-specific ΔG_HX_ of the protein.

The fourth stage of the program has a few numbers of steps as follows: First, the program detects all NHs that are hydrogen bonded in the given 3D protein structure, using method described by Stickle et al [66]. According to the method, the hydrogen bond (H-bond) distance should be < = 3.28 Å and bond angles at the acceptor atom (N—O = C) and at the donor atom (O—N–C_α_) should lie between 90°–180° [66]. Second, of the hydrogen bonded NHs, NHs that are located in the regular secondary structural segments of proteins are segregated on the basis of H-bond patterns (a stretch of i to i+3 or i to i+4 H-bonds for helical conformations and a stretch of i+n to j+n H-bonds for sheets, wherein n is 0,2,4,6 and so on.) and torsion angles (Ф, ψ angles for α-helices, 3_10_ helices, parallel β-sheets and anti-parallel β-sheets are (−57±30, −47±30), (−60±30, −30±30), (−119±30, −113±30) and (−139±30, 135±30), respectively). Third, the program generates all possible residue pairs for the NHs (NHs for which ΔG_HX_ are available) and calculates distance in angstrom between the backbone nitrogen atoms of the two residues in each pair. The program then generates a ‘contact order matrix’ in which each pair is assigned either with the value of 1 or 0: the value of 1 is given to a pair when the distance between the two residues is within 7 Å otherwise 0 is given. Fourth, the program groups the residue-pairs such that any pair in a group must have at least another pair having a residue common to each other. The program avoids redundancy in grouping the residue-pairs and generates atomic coordinate files in PDB format for residues in each group/cluster. If OneG finds more than a cluster for a protein, each cluster is distinct from other clusters in terms of structural contexts. Consequently, each cluster is attributed to possible existence of a cryptic intermediate in the unfolding kinetics of the protein. However, two cryptic intermediates, which are distinct in terms of structural context but indistinguishable in terms of free energy coverage, are represented as cooperative unfolding units.

The program reports no possible existence of cryptic intermediates for a protein, if it predicts single cluster. Only under the circumstance, the program is directed, by default, to predict possible existence of metastable states of proteins, which may lead to the overestimation of the ΔG_HX_, whereas cryptic intermediates accumulating in the unfolding kinetics of proteins lead to underestimation of ΔG_U_. The program generates a cluster consisting of all NHs for which ΔG_HX_>ΔG_X_, which is defined as shown, herein.
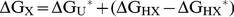
(18)Thus, the ΔG_X_ is the ΔG_U_ corrected for the effects of *cis-trans* proline isomerisation and baselines of melting curves of the proteins. As we discussed in the above paragraph, the residues that are herein grouped into single cluster will be further divided into subgroups on the basis of contact order matrix and any possible cooperative units among the subgroups will then be defined on the basis of ‘free energy coverage’. The reliability of the OneG on the prediction of possible existence of cryptic intermediates/metastable states in the unfolding kinetics of proteins has been tested on the following four proteins: Cytochrome C (1HRC), apocytochrome b_562_ (1APC), Cardiotoxin III (2CRT) and Cobrotoxin (1COD). Of the four proteins, existence of cryptic intermediates in the unfolding kinetics of cytochrome c and apocytochrome b_562_ has been already documented using experimental methods, in the literature [35], [44].

### Chemical denaturation

GdnHCl (guanidine hydrochloride) induced unfolding of ubiquitin dissolved in D_2_O (deuterium oxide) was monitored in the wavelength range from 220 to 230 nm using the AVIV circular dichroism spectrometer. Each spectrum was the average of five scans. The path length was 1 mm and the bandwidth was set to 1 nm. All measurements were made with suitable background corrections. The changes in ellipticity at 222 nm were plotted against the concentration of GdnHCl and the data were fitted to equations 11 & 16 (refer text) to determine the ΔG_U_ of ubiquitin. The data analyses were performed using Kaleidagraph software (Synergy Software, USA).

## Supporting Information

Figure S1 **Flowchart depicting the Stage I of OneG.** Key-steps used to calculate the k_rc_ of NHs in proteins and ΔG_HX_ of proteins are outlined.
(TIF)

Figure S2 **Flowchart depicting the Stage II of OneG.** The flowchart outlines the key-steps used to account the effect of *cis-trans* proline isomerisation on the ΔG_HX_ of proteins.
(TIF)

Figure S3 **Flowchart depicting the Stage III of OneG.** The Flowchart enumerates systematically the various steps to frame two-state model equations for appropriately treating the pre- and post-baselines of melting curves of proteins.
(TIF)

Figure S4 **Flowchart depicting the Stage IV of OneG.** The key-steps involved in the OneG algorithm on predicting cryptic intermediates/higher energy metastable in the unfolding kinetics of proteins under native conditions, are shown.
(TIF)
