# Supplementary figures and images for: OneG: A Computational Tool for Predicting Cryptic Intermediates in the Unfolding Kinetics of Proteins under Native Conditions
Source: PLoS One. 2012 Mar 7;7(3):e32465. doi: 10.1371/journal.pone.0032465 (PMC3296725; doi:10.1371/journal.pone.0032465)

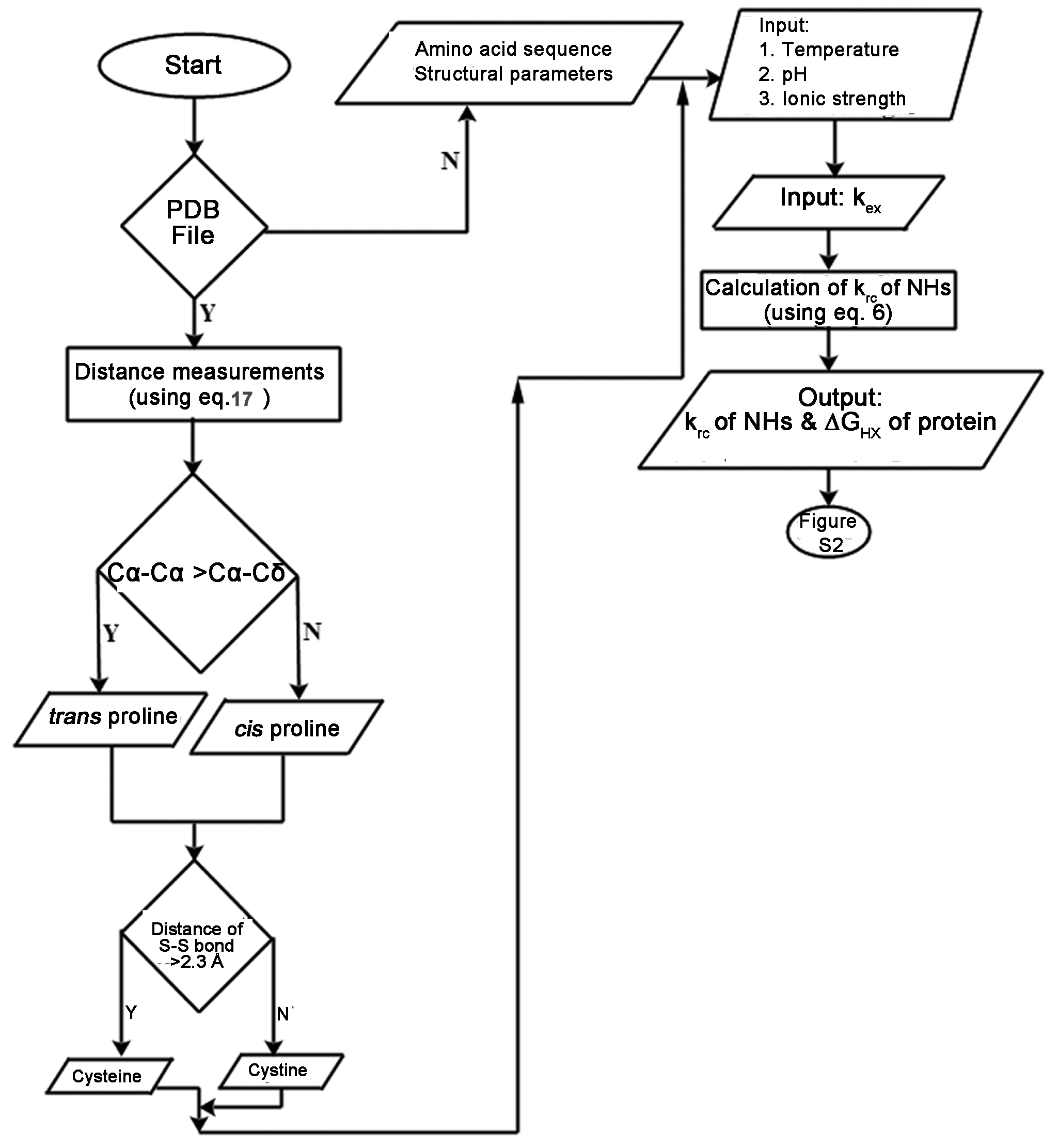

Supplement: Figure S1 — Flowchart depicting the Stage I of OneG. Key-steps used to calculate the krc of NHs in proteins and ΔGHX of proteins are outlined. (TIF) [file pone.0032465.s001.tif]

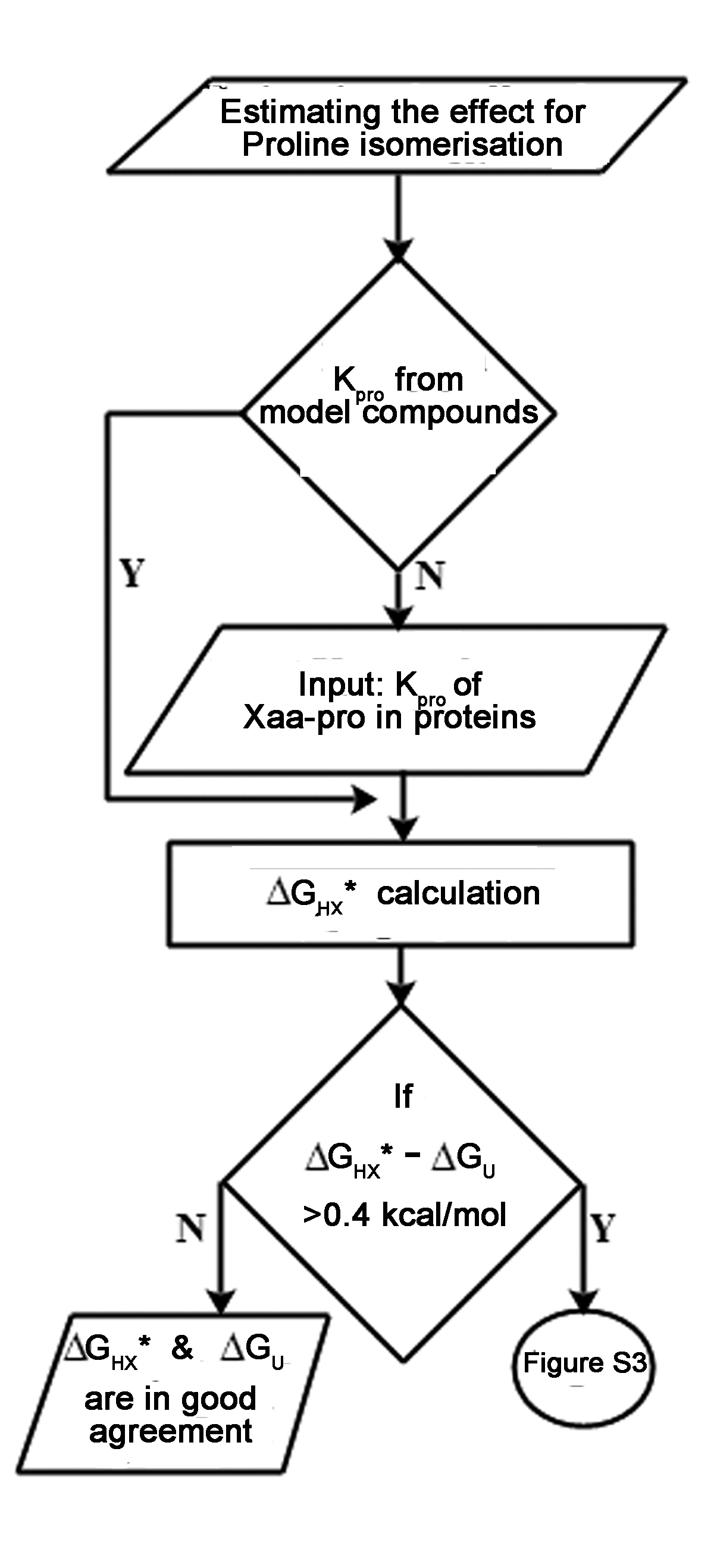

Supplement: Figure S2 — Flowchart depicting the Stage II of OneG. The flowchart outlines the key-steps used to account the effect of cis-trans proline isomerisation on the ΔGHX of proteins. (TIF) [file pone.0032465.s002.tif]

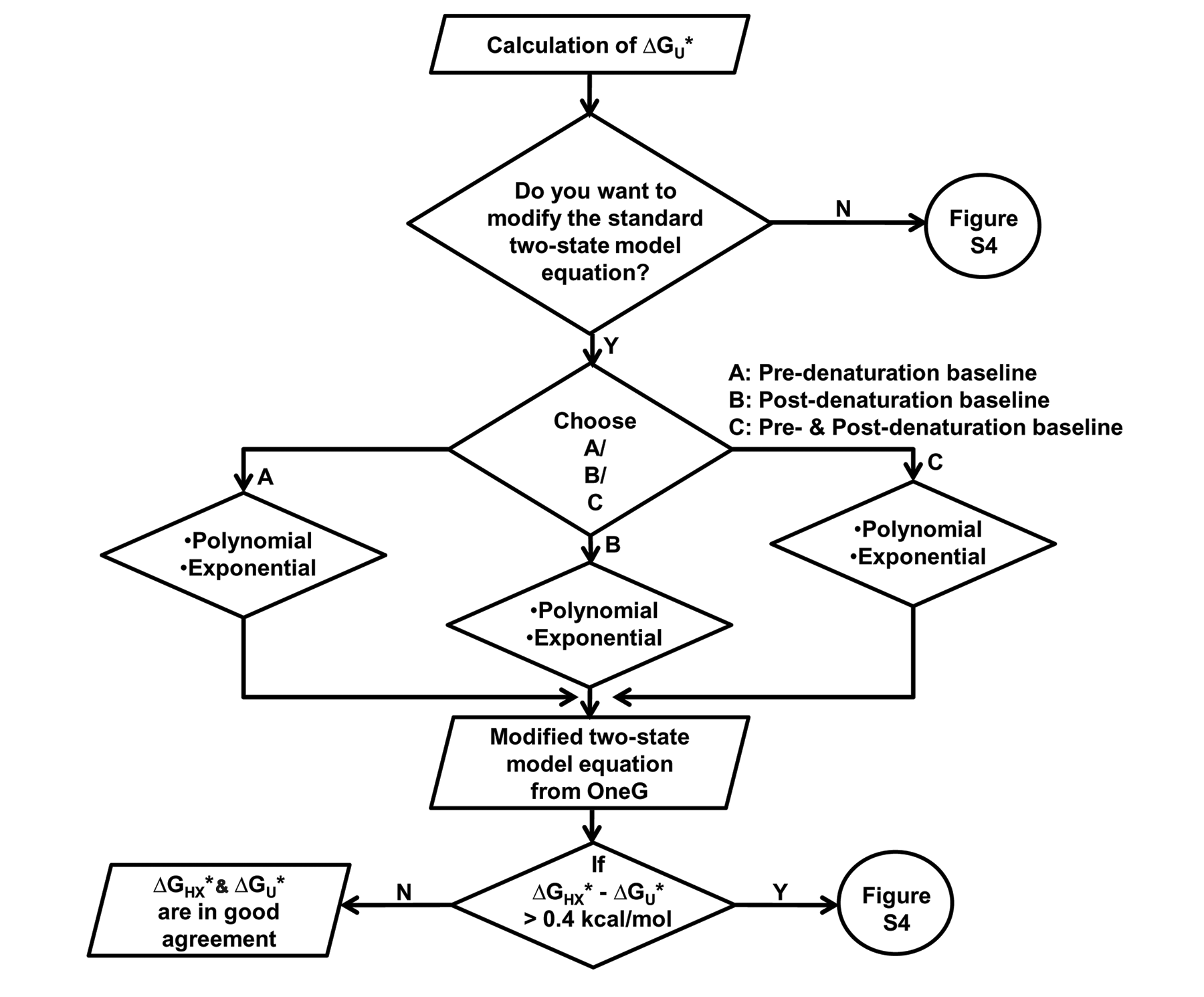

Supplement: Figure S3 — Flowchart depicting the Stage III of OneG. The Flowchart enumerates systematically the various steps to frame two-state model equations for appropriately treating the pre- and post-baselines of melting curves of proteins. (TIF) [file pone.0032465.s003.tif]

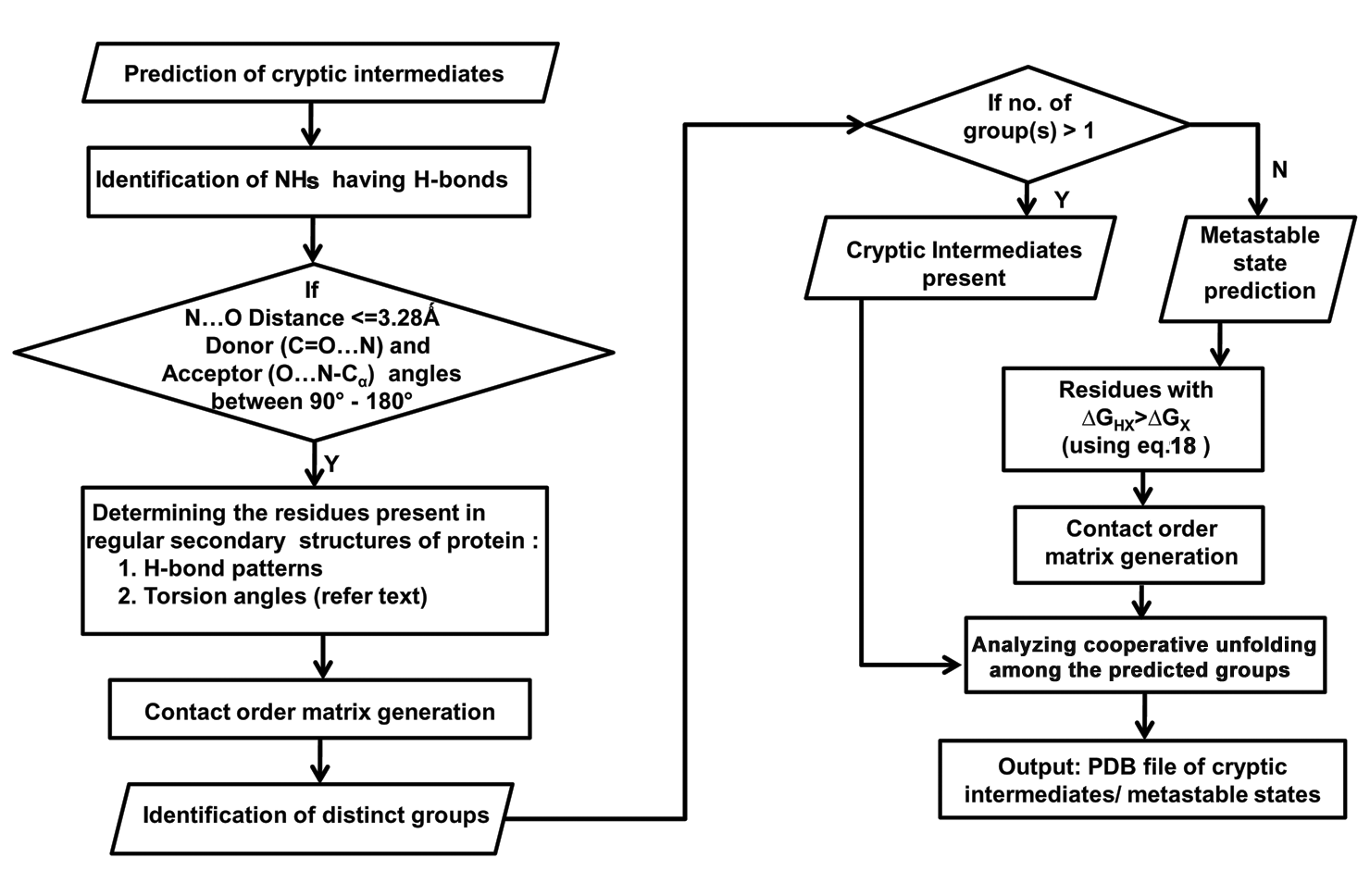

Supplement: Figure S4 — Flowchart depicting the Stage IV of OneG. The key-steps involved in the OneG algorithm on predicting cryptic intermediates/higher energy metastable in the unfolding kinetics of proteins under native conditions, are shown. (TIF) [file pone.0032465.s004.tif]
